# Symmetrized persistency of Bell correlations for Dicke states and GHZ-based mixtures

**DOI:** 10.1038/s41598-021-93786-5

**Published:** 2021-07-12

**Authors:** Marcin Wieśniak

**Affiliations:** 1 Institute of Theoretical Physics and Astrophysics, Faculty of Mathematics, Physics, and Informatics, University of Gdańsk, 80-308 Gdańsk, Poland; 2 International Centre for Theory of Quantum Techologies, University of Gdańsk, 80-308 Gdańsk, Poland

**Keywords:** Optical physics, Quantum physics

## Abstract

Quantum correlations, in particular those, which enable to violate a Bell inequality, open a way to advantage in certain communication tasks. However, the main difficulty in harnessing quantumness is its fragility to, e.g, noise or loss of particles. We study the persistency of Bell correlations of GHZ based mixtures and Dicke states. For the former, we consider quantum communication complexity reduction (QCCR) scheme, and propose new Bell inequalities (BIs), which can be used in that scheme for higher persistency in the limit of large number of particles *N*. In case of Dicke states, we show that persistency can reach 0.482*N*, significantly more than reported in previous studies.

## Introduction

### State-of-art and motivation

Harnessing quantum correlations^1^ can bring us unprecedented possibilities in communication and computation schemes. Core examples are quantum cryptographic key generatifn schemes^2^, which allow an unbreakable message encryption. Their security is guaranteed by an impossibility of cloning of a quantum state, which follows directly from linearity of quantum mechanics. If Eve is trying to intercept a state sent between legitimate users, Alice and Bob, she must destroy quantum coherence, which draws the key generation impossible. In case of distributing entanglement, the same effect occurs by monogamy of entanglement, and in particular, monogamy of violation of CHSH inequality^3^. Simply put, one user (Alice) can violate inequality only with one other partner (Bob or Eve). Security of entanglement-based quantum cryptography was elegantly demonstrated in Ekert’s E91 protocol^4^ .

It is then natural to extend the schemes of quantum cryptographic key distribution to more users, which collaborate to encrypt a message, so that it can be decrypted only by all of them. Such a scenario is called quantum secret sharing^5^, and its security was again linked with violation of multipartite BIs. It is known that, for example, violation of Werner-Wolf-Weinfurter-Żukowski-Brukner (WWWŻB) inequalities^6–8^ is monogamous in a weak sense that only one of the inequalities among overlapping groups of observers can be violated maximally at the time, but not in a stronger sense, where only one can be violated at all. This potentially opens a loophole for an eavesdropper. A further generalization can be based on a subgroup of users, who must collaborate as a qualified majority to unlock a secret.

A relevant protocol is quantum communication complexity reduction (QCCR) in distributed computing. Here, the task is to jointly compute a certain sign function under communication restrictions. Initially, it was shown that GHZ correlations^9^ can reduce one bit of a necessary classical information exchange in certain situations^10^. Subsequently, it was reformulated as a quantum game, in which the goal is to optimize a guess of a 2*N*-bit function, where each user receives two bits and can broadcast only one. It has been shown that there is an advantage in this task once the function expresses a BI and the partners share a corresponding entangled state violating it^11^.

Another concept that we consider here is persistency of quantum correlations. This quantity tells us how many parties must be traced out for a given state to loose its property, e.g. entanglement (denoted as $(P^E(\rho ))$, steerability $(P^S(\rho ))$ (historically first Ref.^12^ gives a slightly different definition), or ability to violate a BI, somewhat misleadingly called “nonlocality” $(P^{NL}(\rho ))$^13^. Hereafter, we will call the last kind persistency of Bell correlations and will be denoted as $P^{\text {Bell}}(\rho )$.

We will consider a stronger, symmetric version of persistency of Bell correlations, $P^{\text {Bell}}_{\text {sym}}(\rho )$. In other words, we will be interested in a number of observers (regardless of their identity) that need to be traced out in order for the observed statistics to have a local realistic description. We take two-fold context for this consideration. For mixtures based on GHZ states, we are mainly interested in the symmetrized quantum communication complexity reduction (sQCCR) game. We will ask what fraction of the total number of players, within an arbitrary ensemble, can achieve an advantage. Thus subsets of a certain cardinally can beat the classical game simultaneously. This consideration could be relevant in environments with significant particle losses or inefficient detectors.

In case of Dicke states^14^, we are more focused on fundamental aspects. In contrast to GHZ states, quantum correlations are present in all their reduced states of more than one qubit. Dicke states are hence strong candidates to show extremely high persistency of Bell correlations.

### Monogamy of Bell correlations

BIs distinguish between quantum-mechanical statistics and those, which permit a local realistic description. Thus, they are essential to recognize advantage Probably the simplest, but very useful one is the CHSH inequality. Consider two users, Alice and Bob, which have a pair of alternative observables, $A_1,A'_1$ for Alice, and $A_2,A'_2$ for Bob. Each of these observables can yield outcomes $+1$ or $-1$. The CHSH inequality then reads

1 $$\begin{aligned} \left\langle {\textit{B}}_{12}\right\rangle =\left\langle A_1\otimes (A_2+A'_2)\right\rangle +\left\langle A'_1\otimes (A_2-A'_2)\right\rangle \le _{LR}2, \end{aligned}$$

where $\le _{LR}$ means that the inequality holds for local realistic theories. The quantum mechanical mean value may reach $2\sqrt{2}\approx 2.82$.

Now, consider a third observer, Eve, which also can has a choice of two local observables $A_3,A'_3$. It has been shown in Ref.^15^ that when Bob performs a CHSH experiment simultaneously with Alice and Eve, only one of respective inequalities can be violated:

2 $$\begin{aligned} \left\langle {\textit{B}}_{12}\right\rangle ^2+\left\langle {\textit{B}}_{23}\right\rangle ^2\le 8 \end{aligned}$$

or in a weaker form^16,17^

3 $$\begin{aligned} \left| \left\langle {\textit{B}}_{12}\right\rangle \right| +\left| \left\langle {\textit{B}}_{23}\right\rangle \right| \le 4 \end{aligned}$$

This result is crucial for the security of quantum cryptographic key distribution. When Eve entangles with legitimate users Alice and Bob, she must decrease quantum correlations between them. At the point Eve knows as much about the generated key as Alice and Bob, the CHSH inequality between the latter becomes satisfied.

The core of the proof lies in the necessary and sufficient condition for violating the inequality. Since we use only two observables per side, we can choose them to be strictly real,

4 $$\begin{aligned} A_i= \, & {} (\sigma _x,\sigma _y,\sigma _z){\vec {c}}_i \\ A'_i= \, & {} (\sigma _x,\sigma _y,\sigma _z){\vec {c}}'_i \\ {\vec {c}}_i=\, & {} \left( \begin{array}{c}\cos \beta _i\\ 0\\ \sin \beta _i\end{array}\right) , \\ {\vec {c}}'_i=\, & {} \left( \begin{array}{c}-\sin \beta _i\\ 0\\ \cos \beta _i\end{array}\right) \end{aligned}$$

(hereafter, the second component is omitted). This choice of observables allows us to consider the reduced state to be strictly real, since the introduction of the nontrivial imaginary part would give the effect of state mixing. As observables of, say, Bob, are shared in both BIs, without a loss of generality strict realness can apply also to observable the third observer and reduced state between the Alice and Eve.

Consider two observers. Notice that $A_1+A'_1=2\cos \beta _1(\sigma _x,\sigma _z){\vec {d}}_1$ and $A_1-A'_1=2\sin \beta _1(\sigma _x,\sigma _z){\vec {d}}'_1$, where ${\vec {d}}_1\perp {\vec {d}}'_1$ and $|{\vec {d}}_1|=|{\vec {d}}'_1|=1$. Thus, introducing $T_{ij}=\left\langle \sigma _i\otimes \sigma _j\right\rangle$, we get that

5 $$\begin{aligned}&\left\langle {\textit{B}}_{12}\right\rangle \\ &\quad =2\left( \begin{array}{cc}T_{xx}&{}T_{xz}\\ T_{zx}&{}TT_{zz}\end{array}\right) \left( \cos \alpha _2{\vec {d_1}}\otimes {\vec {c_2}}+\sin \alpha _2{\vec {d'_1}}\otimes {\vec {c'_2}}\right) , \end{aligned}$$

where ${\vec {c}}_2({\vec {c}}'_2)=\cos \alpha _2{\vec {d}}_2+(-)\sin \alpha _2{\vec {d}}_2$. We now employ the Cauchy-Schwartz inequality, $\left| {\vec {A}}\cdot {\vec {B}}\right| \le \sqrt{|{\vec {A}}|^2|{\vec {B}}|^2}$, where ${\vec {A}}=(T_{xx},T_{xz},T_{zx},T_{zz})^T$ and ${\vec {B}}=\cos \alpha _2{\vec {d_1}}\otimes {\vec {c_2}}+\sin \alpha _2{\vec {d'_1}}\otimes {\vec {c'_2}}$. Obviously $|{\vec {B}}|^2=1$, thus

6 $$\begin{aligned} \left\langle {\textit{B}}_{12}\right\rangle ^2\le 4(T_{xx}^2+T_{xz}^2+T_{zx}^2+T_{zz}^2). \end{aligned}$$

**Figure 1:**
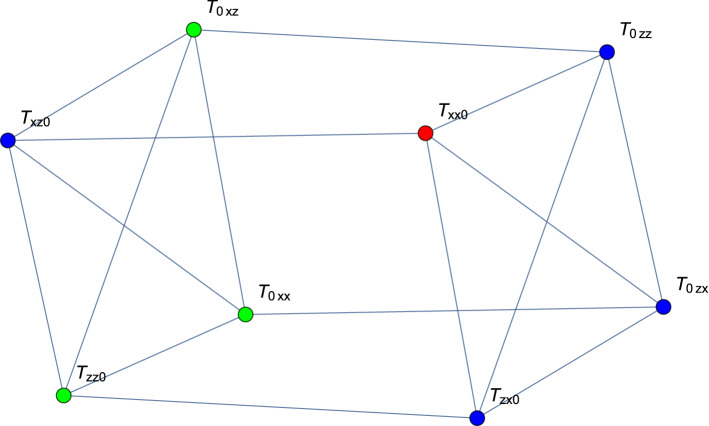
Anticommutativity graph for two CHSH inequalities with one common observer. We can assign value “1” to one of the vertices (red), which forces four other to take value “0” (blue). This will leave three vertices with unassigned value (green).

In this case, the equality can be attained, as we have enough free parameters to conduct the Schmidt decomposition of the used sector of the correlation tensor. Going back to the three-user scenario we get

7 $$\begin{aligned}&\frac{1}{4}\left( \left\langle {\textit{B}}_{12}\right\rangle ^2+\left\langle {\textit{B}}_{23}\right\rangle ^2\right) \\ &\quad \le T_{xx0}^2+T_{xz0}^2+T_{zx0}^2+T_{zz0}^2 \\ &\qquad + T_{0xx}^2+T_{0xz}^2+T_{0zx}^2+T_{0zz}^2, \end{aligned}$$

where $T_{xx0}=\left\langle \sigma _x\otimes \sigma _x\otimes \sigma _0\right\rangle$, etc. Let us now use the methods presented in Refs.^18,19^. We create a graph with 8 vertices associated with the operators, means of which enter Eq. (7), and connect them if they anticommute. We get a cuboid, in which two opposite faces have vertices connected on diagonals, as depicted in Fig. 1. Next, we assign 0s and 1s to the vertices in such a way that no pair of 1s can be connected with an edge. Assigning 1 to any vertex eliminates four connected with it, and the remaing three are in a clique, so only one other 1 can be distributed among them. We thus get

8 $$\begin{aligned} T_{xx0}^2+T_{xz0}^2+T_{zx0}^2+T_{zz0}^2\quad +\quad T_{0xx}^2+T_{0xz}^2+T_{0zx}^2+T_{0zz}^2\quad \le 2, \end{aligned}$$

meaning that once $\left| \left\langle {\textit{B}}_{12}\right\rangle \right|$ goes above 2, $\left| \left\langle {\textit{B}}_{23}\right\rangle \right| \le 2$ cannot be violated and vice versa. In this fashion, we can investigate if the strong monogamy relations hold for other inequalities, in particular, WWWŻB BIs. A rule of thumb is that if all subsets of observers have non-zero overlap, the bound of the sum is $2^{N_0}$, $N_0$ being the cardinality of the largest of these subsets. This happens when parties from this subset share a GHZ state, and hence other parties must be uncorrelated.

**Table 1:** Values for estimating $N_0=a L+b$, above which a WWWŻB inequality can be violated.

M	a	b
1	3	1
2	2.5776	2.8083
3	2.4043	3.6349
4	2.3325	6.4408

1/*a* is a ratio between asymptotic $P^{\text {Bell}_{sym}}$ and *N*.

Now, consider the case of five parties labelled as A, B, C, D, and E. A, and E measure $\frac{1}{\sqrt{2}}(\sigma _x\pm \sigma _y)$, while B, C, and D measure $\sigma _x$ or $\sigma _y$. In one half of the runs, A,B,C, and D receive a GHZ state, and E receives the white noise, in the other half the roles of A and E are interchanged. If the first four observers shared a pure GHZ state, they would get the violation of a Mermin-Ardehali-Belinskii-Klyshko (MAKB) inequality^20–22^ by factor $2\sqrt{2}$, but the state has effectively 50% of noise. Thus, both ensembles, $\{$A, B, C, D$\}$ and $\{$B, C, D, E$\}$, can simultaneously violate a MAKB inequality. Hence we have

9 $$\begin{aligned} P^{\text {Bell}}\left( 1/4\left( \left| GHZ_4\right\rangle \left\langle GHZ_4\right| \otimes {\mathbb{1}}_{2\times 2} +{\mathbb{1}}_{2\times 2}\otimes \left| GHZ_4\right\rangle \left\langle GHZ_4\right| \right) \right) =2, \end{aligned}$$

where

10 $$\begin{aligned} |GHZ_l\rangle = \frac{1}{\sqrt{2}}\left( \left| 0\right\rangle ^{\otimes l}+\left| 1\right\rangle ^{\otimes l}\right) . \end{aligned}$$

In general we shall use “greater than” sign, rather than “equal to” for persistency of Bell correlations, as we cannot claim that we use the best inequalities. In this case, we clearly use the optimal inequality.

We will investigate a symmetrization of this scenario.

### Quantum communication complexity reduction

Here we briefly recall the idea behind the quantum advantage in communication complexity reduction problems in distributed computing related to BIs. This link was established in Ref.^11^, but it is only one-way^23^. Originally, it was shown that when using a GHZ state, a certain function can be computed if users exchange one bit less in total^10^, but Ref.^11^ introduced the following probabilistic interpretation. Imagine *N* users, whose task is to jointly calculate a certain dichotomic function. Each user receives two random variables from a dealer: a random bit $y_i=\pm 1$, with promise of $P(y_i=+1)=1/2$, and $x_i$, which can be from any set, and the joint distribution of $x_i$s is promised,

11 $$\begin{aligned} P(x_1,\ldots ,x_N)=\frac{|g(x_1,\ldots ,x_N)|}{\sum _{x_1,\ldots ,x_N}|g(x_1,\ldots ,x_N)|} \end{aligned}$$

(or with integrals in the denominators). Now, a user can perform an arbitrary local action, but must return (broadcast) one bit. From these bits they guess the value of function

12 $$\begin{aligned} F(x_1,\ldots ,x_N,y_1,\ldots ,y_N)=y_1\ldots y_N\frac{g(x_1,\ldots ,x_N)}{|g(x_1,\ldots ,x_N)|}=\pm 1. \end{aligned}$$

The ultimate task is to yield the correct value of $F(x_1,\ldots ,x_N,y_1,\ldots ,y_N)$ in as many cases as possible.

Obviously, broadcasted bits must be contain information about $y_i$s, since omitting any one of them completely destroy the correlation between the actual and the anticipated value. Then, if all $g(x_1,\ldots ,x_N)$s are of the same sign, or 0, the task trivializes. If the sign varies, in the classical case the users are limited to broadcast $y_if_i(x_i)=\pm 1$. Thus they are restricted to local deterministic predictions. When entangled state $\left| \Psi \right\rangle$ of *N* qubits is distributed among them, they can, however, make a measurement on a qubit they hold dependent on $x_i$, obtain result $m_i$, and broadcast $y_im_i$. Then, if $g(x_1,\ldots ,x_N)$s are coefficient of a BI violated by $\left| \Psi \right\rangle$, the users take benefit from quantum correlations, and get a more efficient estimation of $F(x_1,\ldots ,x_N,y_1,\ldots ,y_N)$. Thus, this variant is will be hereafter QCCR game.

We will be interested in a bit modified variant of this scheme, the sQCCR game. We will still have *N* users, but we demand that only *k* is trying to estimate the function. Our restriction, though, is that this could be any subset of *k* users, or, equivalently, each such group tries to estimate the function independently. For the sQCCR game, we additionally require that the marginal probabilities are symmetric under permutations of parties, i.e.,

13 $$\begin{aligned} P(x_{\pi _1},x_{\pi _2},\ldots ,x_{\pi _k})=\sum _{x_{\pi _{k+1}},\ldots ,x_{\pi _N}}P(x_{\pi _1},x_{\pi _2},\ldots ,x_{\pi _N}), \end{aligned}$$

where $(\pi _1,\ldots ,\pi _N)$ is an arbitrary permutation of $(1,\ldots ,N)$.

We will also consider violation of a BI under such restrictions. The difference between a mere BI violation and QCCR scheme lies in the demand that in latter case, the measurements settings are distributed with a known probability distribution. It might be impossible to find a distribution that has a desired one as all marginals of *k*th order. In case of *BI*s, user draw the measurements settings locally and independently to close the common cause loophole, so frequency of their appearance only affects the trust level of average values. Most optimally, a flat distribution is used.

## Results

### Symmetrized persistency of Bell correlations for GHZ-based mixtures

First, let us discuss the case of GHZ states. Obviously, any quantum advantage for $N-L<N$ of *N* users is not possible for pure states, as any reduced state is fully separable. We thus need to use a symmetrized mixture,

14 $$\begin{aligned} \rho =\frac{1}{2^{L}N!}\sum _{\Pi }\Pi \left( \left| GHZ_{N-L}\right\rangle \left\langle GHZ_{N-L}\right| \otimes {\mathbb{1}}_{2^{L}\times 2^{L}}\right) , \end{aligned}$$

where $\sum _{\Pi }\Pi (\cdot )$ denotes the sum over all permutations of parties. We refer to these states as GHZ based mixtures.

Let us start with MAKB inequalities, which are obtained in an iterative way. Each observer has choice two observables, $A_i$ and $A'_i$. The Bell expressions are

15 $$\begin{aligned}B_2(A_1,A'_1,A_2,A'_2) &=\frac{1}{2}(A_1\otimes (A_2+A'_2)+A'_1\otimes (A_2-A'_2)), \\ &\qquad B_{i+1}(A_1,A'_1,\ldots ,A_{i+1},A'_{i+1}) \\ & = \frac{1}{2}((A_{i+1}+A'_{i+1})\otimes B_i(A_1,A'_1,\ldots .,A_i,A'_i)\\ &\quad +(A_{i+1}-A'_{i+1})\otimes B_i(A'_1,A_1,\ldots ,A'_i,A_i) \end{aligned}$$

The maximal local realistic values of $B_N$ are $2^{\frac{N-1}{2}}$ for odd *N* and $2^{\frac{N}{2}}$ for even. If we take $A_i=\cos (2\pi \alpha _i) \sigma _x+\sin (2\pi \alpha _i) \sigma _y$ and likewise for $A'_i$s, we get

16 $$\begin{aligned} \left\langle GHZ_N\right| A_1\otimes \cdots \otimes A_N\left| GHZ_N\right\rangle =\cos \left( 2\pi \sum _i\alpha _i\right) . \end{aligned}$$

For odd *N* we have $2^{N-1}$ average values, and with choice of observables $A_i=\sigma _x$, $A'_i=\sigma _y$ all of them have modulo 1 and a sign corresponding to the respective terms in the Bell expression. Thus the maximal quantum mechanical value is $2^{N-1}$, and the quantum-to-classical ratio (QCR), the ratio between the maximal quantum and classical values, is $2^{(N-1)/2}$. For even *N*, again, all signs are matched, but the optimal modulo is $1/\sqrt{2}$ an QCR is still $2^{(N-1)/2}$. A symmetrized optimal choice of observables is $\alpha _i=\frac{1}{8N}$ and $\alpha '_i=\frac{2N+1}{8N}$.

In case of GHZ states, even stronger inequalities were found. They utilize a continuum of local observables, i.e. $\alpha _i$ will take an arbitrary value. Naturally, any feasible implementation of these inequalities will utilize a finite number of uniformly distributed vales of $\alpha _i$^24^. The coefficiens are given by the values of the quantum correlation function and the QCR is $\frac{1}{2}\left( \frac{\pi }{2}\right) ^N$.

### Symmetrized persitency of Bell correlations in QCCR protocols

As we have seen, for GHZ-based mixtures, QCR grows exponentially with the number of parties, while the symmetrization causes only a polynomial decay of correlation. Thus for any value *L* there is some value of *N*, above which any *L* users can be traced out.

For MAKB inequalities and geometrical BIs (GBIs)^25,26^ QCR and $(N-L)$-partite GHZ states behaves like

17 $$\begin{aligned} QCR(N-L)=b\times a^{N-L}, \end{aligned}$$

where $a=\sqrt{2}$, $b=1/\sqrt{2}$ for MAKB inequalities and $a=\pi /2$, $b=1/2$ for GBIs. Let us now extend and symmetrize the state so that any $N-L$ users can violate a BI,

18 $$\begin{aligned} \left( \begin{array}{c}N\\ N-L\end{array}\right) ^{-1}b\times a^{N-L}>1, \end{aligned}$$

which is the condition for $P^{\text {Bell}}_{\text {sym}}>L$. $P^{\text {Bell}}_{\text {sym}}\ge 2$ occurs for $N=9$ in case of MAKB inequalities and $N=7$ for GBIs. Let us now study the asymptotic behavior with $N\rightarrow \infty$ by referring to the Stirling approximation and introducing $\gamma =L/N$.

19 $$\begin{aligned} 0< & {} H(\gamma )-\gamma \log _2{a}-\frac{\log _2{b}}{N}, \\ H(x)= & {} -x \log _2{x}-(1-x)\log _2(1-x). \end{aligned}$$

Note that $\frac{\log _2{b}}{N}$ is neglible for large *N*. We are thus left with

20 $$\begin{aligned} H(\gamma )>\gamma \log _2{a}. \end{aligned}$$

We can now find the ratio of parties that must be preserved. This cannot be done analytically, but is guaranteed to happen for any *a*: $H(0)=0$,$\left. \partial H(x)/\partial x\right| _{x=0}=+\infty$, $H(1-x)=H(x)$, and $H(x)\le 1$. For $a=\sqrt{2}$ Eq. (20) is satisfied for $\gamma <\gamma _{CRIT}=0.905118$, whereas $a=\pi /2$ gives $\gamma _{CRIT}=0.867227$.

Thus, in the limit of large *N* up to 13.2% of users can be replaced by others in the protocol. However, gaining an advantage in communication complexity reduction scheme requires an extra discussion. As we have mentioned above, in case of a mere Bell test distribution of measurement settings is largely irrelevant, and it is even desired to be uniform, in the QCCR scheme this distribution encodes the BI. For example, for an even number of parties with MAKB inequalities, we use all combinations of observables with equal weights, but only a half of them for odd. To enjoy the benefit in QCCR we need to have even *k*. Otherwise, after replacing one of the partners with another one we would not be able to recreate the distribution.

A similar problem arises with GBIs. However, this can be easily fixed by introducing new GBIs, in which observables from the *x*–*y* plane (with eigenvalues $\pm 1$). Thus the quantum mechanical part reads

21 $$\begin{aligned} Q_N= & {} \int _0^1d\alpha _1\ldots \int _0^1d\alpha _N\text {sign}\left( \cos \left( 2\pi \sum _i\alpha _i\right) \right) \cos \left( 2\pi \sum _i\alpha _i\right) \\= & {} \int _0^1d\alpha _1\ldots \int _0^1 d\alpha _N\left| \cos \left( 2\pi \sum _i\alpha _i\right) \right| \\= & {} \frac{2}{\pi }. \end{aligned}$$

The optimal classical part is equal to

22 $$\begin{aligned} C_N=2^N\int _{-N/4}^{(-N+1)/4}d\alpha _1\int _{0}^{1/2}d\alpha _2\ldots \int _{0}^{1/2}d \alpha _N\text {sign}\left( \cos \left( 2\pi \sum _i\alpha _i\right) \right) \end{aligned}$$

and $\{C_2,\,C_3,\,C_4,\,C_5,\,C_6,\,C_7,\ldots \}\,=\{1/2,\,1/3,\,5/24,\,2/15,\,61/720,\,17/315\,\ldots \}$. We have

23 $$\begin{aligned} \lim _{N\rightarrow \infty }\frac{C_{N-1}}{C_N}=\frac{\pi }{2}. \end{aligned}$$

and the convergence is exponential. The first instance of persistency of $P^{\text {Bell}}_{\text {sym}}\ge 2$ occurs for $N=7$, ${Q_6}/(7{C_6})=1440/(427\pi )\approx 1.07346$.

### Symmetrized persistency of Bell correlations for Dicke states

Dicke state family is exemplary for studying persistency of Bell correlations,

24 $$\begin{aligned} \left| D_{N,M}\right\rangle = \left( \begin{array}{c}N\\ M\end{array}\right) ^{-\frac{1}{2}}\Pi (|0\rangle ^{\otimes M} |1\rangle ^{\otimes N-M}), \end{aligned}$$

where $\Pi (\cdot )$ denotes the sum over all permutations of parties. In particular, Refs.^14,27–30^ studied $P^{\text {Bell}}_{\text {sym}}$ for W states ($\left| W_N\right\rangle =\left| D_{N,1}\right\rangle$). In particular, the Authors of Ref.^30^ considered BIs involving correlation between subsets of observers.

To set a context of this section, let us remind the findings from Ref.^30^. The Authors have given the upper bound for persistency for all states symmetric with respect to permutation of particles of N/2. The lower bound was given by

25 $$\begin{aligned} P>N'-N+1, \end{aligned}$$

where $N'=\left\lfloor N/(1-p_{CRIT})\right\rfloor$, $\lfloor \cdot \rfloor$ is the floor function and $p_{CRIT}$ is the critical probability below which state

26 $$\begin{aligned} \rho (N,p)=(1-p)\left| W_N\right\rangle \left\langle W,N\right| +p\left| 0\right\rangle ^{\otimes N}\left\langle 0\right| ^{\otimes N} \end{aligned}$$

violates a Bell inequality with *m* settings per side. Ref.^30^ shows this bound for $m=2,\ldots ,6$ and *N* up to 16. As a result, they find two-setting-per-side Bell inequalities, for which the persistency of Bell correlations reaches 0.4*N* in the limit of large *N*.

In this work we limit ourselves to WWWŻB inequalities. The respective Bell operators read

27 $$\begin{aligned} B'_N= \, & {} \frac{1}{2^N}\sum _{s_1,\ldots ,s_N=\pm 1}S(s_1,\ldots ,s_N) \\ &\times (A_1+s_1A'_1)\otimes \cdots \otimes (A_N+s_NA'_N) \\\le \, & {} 1, \\ S(s_1,\ldots ,s_N)=\, & {} \pm 1. \end{aligned}$$

We thus have a choice of $2^{2^N}$ different operators, one for each sign function $S(s_1,\ldots ,s_N)$ (naturally, they can be either trivial, or trivially related amongst them).

The necessary condition for violation of WWWŻB inequalities is that there exists such a choice of local directions *x* and *z*, that

28 $$\begin{aligned} \sum _{i_1=x,z}\ldots \sum _{i_N=x,z}T^2_{i_1i_N}>1. \end{aligned}$$

The problem with violation of WWWŻB inequalities with Dicke states is that there is a violation gap. That is to say, there is a certain range of white noise admixture, for which the state satisfies condition (28), but still does not violate any inequality from this family. However, this gap is relatively small [typically less than 1% of the sum in Eq. (28)], hence the sum is hence a good and fast indicator of potency for violation. Additionally, the same condition becomes necessary and sufficient for violation of inequalities with more settings per side under the restriction that the observables lie in the *x*–*y* plane^31^.

The partial trace of the Dicke state over *L* parties is

29 $$\begin{aligned}&\rho _{N,M,L} \\ &\quad =\text {Tr}_L(\left| D_{N,M}\right\rangle \left\langle D_{N,M}\right| ) \\ &\quad = \left( \begin{array}{c}N\\ M\end{array}\right) ^{-1}\sum _{l=0}^L\left( \begin{array}{c}L\\ l\end{array}\right) \left( \begin{array}{c}N-L\\ M-l\end{array}\right) \left| D_{N-L,M-l}\right\rangle \left\langle D_{N-L,M-l}\right| . \end{aligned}$$

In the next step we calculate

30 $$\begin{aligned} \Sigma (N,M,L) &=\sum _{i_1=x,z}\ldots \sum _{i_{N-L}=x,z}T^2_{i_1\ldots i_{N-L}}(N,M,L) \\ & =\sum _{i_1=x,z}\cdots \sum _{i_{N-L}=x,z}\text {Tr}\rho _{N,M,L}(\sigma _{i_1} \otimes \cdots \otimes \sigma _{i_{N-L}}) \\ & =\sum _{a=0,2,\ldots ,2L}\left( \begin{array}{c}N-M\\ a\end{array}\right) \\ &\qquad \left( \sum _{b=0,2,\ldots ,a} \left( \begin{array}{c}N-M\\ b\end{array}\right) \left( \begin{array}{c}M\\ L-b\end{array}\right) \left( \begin{array}{c}b\\ b/2\end{array}\right) \left( \begin{array}{c}N-b\\ M-b/2\end{array}\right) (-1)^{M-b/2}\right) ^2. \end{aligned}$$

Note that this is a straight-forward generalization of states given by Eq. (26).

These data are then interpolated to function $\Sigma _{M,L}(N)$ and equation $\Sigma _{M,L}(N_0)=1$ is solved for $N_0$. The values of $N_0$ are given in Table 1.

If the results hold the pattern for larger *M*, we shall have $a\approx 2.0925 1+1/M$. We thus have $P^{\text {Bell}}_{\text {sym}}\ge 2$ for $(N,M)\in \{(5,1),(6,2),(8,3),(9,4)\}$. As we can see, in the limit of both large *N* and large *L* we estimate that $P^{\text {Bell}}_{sym}\ge 0.482N$ for $M\rightarrow N/2$

Eq. (30) allows us to further investigate the estimates of the persistency for Dicke states with $M=N/2$. For very large *N* we have

31 $$\begin{aligned} \Sigma (N,N/2,L)\approx e^{c(L/N)N}. \end{aligned}$$

We have considered the values of *c*(*L*/*N*) with $N=600$ up to $L=0.39N$, $N=1000$ up to $0.4N\le L\le 0.47N$, and $N=3000$ for $L=0.48N$. It turned out that $\sqrt{c(L/N)}$ in the interval of [0.01, 0.48] can be well approximated as $\sqrt{c(L/N)}\approx \sqrt{10}(1.0855-2.2686L/N)$, which becomes negative for $L/N=0.478$, but the approximation breaks down a bit for large $\gamma$. However, we have also studied the value of $\Sigma (N,N/2,0.48)$, which reads 1.00265 for $N=8500$, 1.4097 for $N=9000$ and 2.8139 for $N=10000$.

In case of W states we have studied some particular inequalities. None of them satisfied condition (13). Thus we assume that they are not useful for the sQCCR game.

## Conclusions

We have studied symmetrized Bell correlations persistency for mixtures of GHZ states and Dicke states, especially in the context of their link to quantum communication schemes. We found that mixtures based on GHZ states asymptotically allow for loss of about 9.5% to MAKB inequalities and 13.2% for GBIs.

We have characterized the persistency of Bell correlations of Dicke states with respect to WWWŻB inequalities. We have observed that already for three excitations we have asymptotic persistency higher than 2/5*N*, and in the limit of large number of excitations, we estimate it reach 0.482*N*. We have also demonstrated that Dicke states with half of the qubits excited can violate a Bell inequality even if 0.48 of all parties are traced out, though it would require more than 8000 qubits. Thus, contrary to the remarks in Ref.^30^, many-excitation Dicke states perform significantly better than the reported persistency of Bell correlations for W states, even though we have not tailored BIs for the former.

We have found that in a symmetrized situation described in the current paper users sharing the GHZ states-based mixture can still enjoy the quantum advantage in the sQCCR game in subgroups of up to 0.89*N* users. They can achieve this using even-number-of-parties MAKB BIs, or a variant of GBIs, which asymptotically has quantum-to-classical ratio proportional to $(\pi /2)$
